# Saussureae Radix Attenuates Neuroinflammation in LPS-Stimulated Mouse BV2 Microglia via HO-1/Nrf-2 Induction and Inflammatory Pathway Inhibition

**DOI:** 10.1155/2021/6687089

**Published:** 2021-03-18

**Authors:** You-Chang Oh, Wei Li, Jang-Gi Choi

**Affiliations:** Korean Medicine (KM)-Application Center, Korea Institute of Oriental Medicine, 70, Cheomdanro, Dong-gu, Daegu 41062, Republic of Korea

## Abstract

The activation of microglial cells and their subsequent neuroinflammatory reactions are related to various degenerative brain diseases. Therefore, the regulation of microglial cell activation is an important point for the research of therapeutic agents for treating or preventing neurodegenerative disorders. Saussureae Radix (SR) is the root of *Saussurea lappa* Clarke, and it has been used for a long time as an herbal medicine in East Asia to treat indigestion and inflammation of the digestive system. In previous studies, however, the effect of SR ethanolic extract on microglial cell-mediated neuroinflammation was not fully explained. In this study, we explored the antineuroinflammatory activities and molecular mechanisms of SR in microglial cells stimulated with LPS (lipopolysaccharide). Our results illustrated that SR does not cause cytotoxicity and significantly weakens the production of nitric oxide (NO) and inflammatory cytokines. SR treatment also inhibited the expression of inducible nitric oxide synthase (iNOS) and cyclooxygenase- (COX-) 2, induced heme oxygenase- (HO-) 1, and activated the nuclear factor erythroid 2-related factor 2 (Nrf-2) pathway. In addition, SR significantly repressed the transcriptional activities of the nuclear factor- (NF-) *κ*B and activator protein- (AP-) 1. Furthermore, SR effectively inhibited the phosphorylation of mitogen-activated protein kinase (MAPK) and Janus kinase (JAK)/signal transducer and activator of transcription (STAT). Isolation and high-performance liquid chromatography (HPLC) analysis indicated two major sesquiterpenoids (costunolide and dehydrocostuslactone). These compounds significantly inhibited the production of neuroinflammatory mediators and induced HO-1 expression. These findings show that SR could be a potential candidate for the treatment of inflammation-related degenerative brain diseases.

## 1. Introduction

Microglia are important immune cells, and they play critical roles in the central nervous system (CNS) in the brain. In normal conditions, they are continually scavenging the CNS for abnormal brain cells and pathogens, and they also help regulate synaptic homeostasis [1]. However, activated conditions of microglia give rise to neurotoxic substance production, such as inflammatory mediators and proteins, all of which are implicated in neurodegenerative disorders [2]. LPS acts as a prototypical endotoxin, inducing inflammation, sepsis, and death. LPS is thus commonly used to create *in vitro* models of inflammation [3]. In activated conditions upon LPS stimulation, MAPKs in turn mediate some signal pathways, such as the transcription factors NF-*κ*B and AP-1 [4]. AP-1 and NF-*κ*B are closely connected in the production of inflammatory molecules, including iNOS and cytokines [5]. In addition, the JAK/STAT is another important pathway with critical roles in immune responses via the release of growth factors and proinflammatory cytokines [6]. Accordingly, regulation of the MAPK, NF-*κ*B, AP-1, and JAK/STAT pathways can be important for treating inflammatory diseases.

HO-1 is an enzyme that promotes the decomposition of heme into carbon monoxide (CO) and biliverdin. HO-1 is highly inducible, and it is expressed in many neuronal cells, including HT22 and BV2 cells [7]. In addition, HO-1 inhibits the production of proinflammatory factors, such as NO and inflammatory cytokines [8]. HO-1 and its by-product CO decrease iNOS expression, thereby reducing the level of iNOS-derived NO [9]. HO-1 production is induced by activation of redox-dependent transcription factor Nrf-2 [10]. In the inflammatory process, free Nrf-2 translocates to the nucleus and consequently induces HO-1 production [11]. Thus, some recent studies identified the importance of HO-1 for immunomodulation or anti-inflammatory efficacy.

SR has long been used as a medicinal herb to treat various diseases of the digestive system. Previous studies reported that SR can ameliorate ethephon-induced reproductive toxicity in rats and alleviate house dust mite-induced atopic-like dermatitis in Nc/Nga mice [12, 13]. Also, another review study showed that SR exhibits anticancer, anti-inflammatory, antiulcer, and cholagogic effects [14]. However, the influence of SR and its molecular mechanisms on neuroinflammation in microglial cells remain unknown. Thus, the present study investigated the inhibitory effect of SR on neuroinflammation and its mechanisms using BV2 microglial cells upon LPS stimulation. To evaluate the antineuroinflammatory efficacy of major constituents in SR, we isolated two major sesquiterpenoids, costunolide (1) and dehydrocostuslactone (2). Furthermore, two isolated compounds were also investigated quantitatively using HPLC analysis, and we explored its antineuroinflammatory efficacy.

## 2. Materials and Methods

### 2.1. Materials and Reagents

Dulbecco's modified Eagle's medium (DMEM), fetal bovine serum (FBS), and antibiotics were obtained from Hyclone (Logan, UT, USA). LPS, dexamethasone, bovine serum albumin (BSA), and dimethyl sulfoxide (DMSO) were purchased from Sigma-Aldrich (St. Louis, MO, USA). 100 mm culture dishes and 6–96-well plates were obtained from Sarstedt (Nümbrecht, Germany). A cell-counting kit (CCK) was purchased from Dojindo (Kumamoto, Japan). Enzyme-linked immunosorbent assay (ELISA) antibody sets were obtained from eBioscience (San Diego, CA, USA). Various primary antibodies and horseradish peroxidase- (HRP-) conjugated secondary antibodies were purchased from Cell Signaling Technology, Inc. (Boston, MA, USA) and Novus Biologicals (Centennial, CO, USA). The Alexa Fluor 488-conjugated anti-rabbit secondary antibody was obtained from Invitrogen (Carlsbad, CA, USA). An RNA extraction kit was obtained from iNtRON Biotech (Daejeon, Korea). Oligonucleotide primers for real-time reverse transcription-polymerase chain reaction (RT-qPCR), DNA synthesizing kits, and AccuPower® 2x GreenStar qPCR Master Mix were obtained from Bioneer (Daejeon, Korea). SR ethanolic extract (used 100% ethanol) was obtained from the Korea Plant Extract Bank (Ochang, Korea).

### 2.2. Cell Culture, Test Drug Treatment, and Stimulation

BV2 cells were grown in DMEM (contains 1% antibiotics and 10% FBS) and incubated in a humidified 5% CO_2_ atmosphere at 37°C. To stimulate the microglia, LPS (100 ng/mL) was used in the presence or absence of SR (1, 10, 50, or 100 *μ*g/mL), costunolide (1, 10, or 25 *μ*M), dehydrocostuslactone (1, 5, or 10 *μ*M), or DMSO (0.1%, vehicle control (VC)).

### 2.3. Cell Viability Test

The potential cytotoxicity of SR was examined by CCK assay. BV2 cells were seeded and preincubated for 18 h, and various concentrations of SR, DMSO, or LPS were added to the cells. After incubation for 24 h, the CCK solution was added and incubated for an additional 1 h. Cell viability was calculated from the optical density at 450 nm using an ELISA reader.

### 2.4. Analysis of NO Secretion

NO levels were examined by measuring nitrite concentrations in the culture medium. BV2 cells were plated, preincubated with SR, costunolide, dehydrocostuslactone, or DMSO for 1 h, and stimulated with LPS for 24 h. Then, 100 *μ*L of the Griess reagent was added to each well. After incubation at room temperature for 5 min, absorbance was measured at 570 nm using an ELISA reader.

### 2.5. Cytokine Determination

For ELISA, BV2 cells were seeded and incubated for 18 h. The cells were pretreated with several concentrations of SR, costunolide, dehydrocostuslactone, or DMSO for 1 h, then stimulated with LPS for an additional 6 h. The inflammatory cytokine levels of the culture medium were measured by the ELISA antibody set in accordance with the manufacturer's protocol.

### 2.6. Total RNA Extraction and RT-qPCR

Total RNA extraction and cDNA synthesis were carried out using easy-BLUE™ RNA extraction kits (iNtRON Biotech) and AccuPower® CycleScript RT PreMix (Bioneer), respectively, and the methods of previous studies were referred [15]. The experimental setting, specific methods, and PCR conditions for RT-qPCR referred to our previous research methods [15]. The sequence of oligonucleotide primers used in this study is shown in Table 1 [15]. The amplification and analysis were performed using a QuantStudio 6 Flex Real-time PCR System (Thermo Fisher Scientific, Rockford, IL, USA), and each sample was compared by the relative CT method. The results of RT-qPCR were presented as gene induction fold, which was calculated using the internal control.

### 2.7. Preparation of Whole-Cell, Cytosolic, and Nuclear Extracts

To obtain whole-cell lysates, pellets were resuspended in the radioimmunoprecipitation assay lysis buffer (Millipore, Bedford, MA, USA) containing protease and phosphatase inhibitors. Cytosolic and nuclear fractions were isolated using NE-PER™ nuclear and cytoplasmic extraction reagents (Thermo Fisher Scientific) as described by the manufacturer.

### 2.8. Western Blotting Analyses

Total proteins were normalized using Bradford's method [16]. The proteins were separated using sodium dodecyl sulfate-polyacrylamide gel electrophoresis and transferred to a polyvinylidene fluoride membrane (Millipore, Bedford, MA, USA). After blocking nonspecific binding sites using 3% BSA, membranes were incubated with each primary antibody at 4°C overnight. The membranes were subsequently incubated with each HRP-conjugated secondary antibody. Protein levels were quantified using a ChemiDoc™ Touch Imaging System (Bio-Rad). The information about the various primary and secondary antibodies is listed in Table 2.

### 2.9. Immunofluorescence Staining

BV2 cells were seeded and incubated overnight. After treatment with SR and LPS, BV2 cells were incubated for 1 (NF-*κ*B p65) or 3 h (Nrf-2). Cells were washed three times with phosphate-buffered saline (PBS) and fixed in 4% paraformaldehyde for 30 min at room temperature. After blocking, immobilized cells were incubated with each primary antibody overnight at 4°C, washed three times with PBS, and incubated with the Alexa Fluor 488-conjugated secondary antibody. Cells were incubated with 4,6-diamidino-2-phenylindole (DAPI) for 10 min at room temperature and observed under a fluorescence microscope [17].

### 2.10. Plant Material

Dried SR was kindly provided from Bomyeong Herbal Market, Seoul, in 2016. Its scientific name was identified by one of the authors (Dr. Wei Li). A voucher specimen (ID-160078) was deposited at the Herbarium of Korean Medicine (KM)-Application Center, Korea Institute of Oriental Medicine, Republic of Korea.

### 2.11. Extraction and Isolation

The dried SR (500.0 g) was reflux extracted three times using EtOH (1.5 L). The total extraction (80.0 g) of EtOH was suspended in deionized water and partitioned with EtOAc and water fraction, yielding EtOAc (1A, 38.0 g) and water (1B, 41.5 g). The EtOAc fraction was subjected to silica gel column chromatography with a gradient of hexane-EtOAc-MeOH (10 : 1 : 0, 9 : 1 : 0, 8 : 1 : 0, 6 : 1 : 0.1, 5 : 1 : 0.1, 4 : 1 : 0.1, 3 : 1 : 0.1, and 2 : 1 : 0.1, with 1.0 L for each step) to give 11 fractions (Fr. 1A-1-1A-11). Fraction 1A-2 was isolated with a gradient of H_2_O-MeOH (1 : 4, 1 : 3, 1 : 2, and 1 : 1 and MeOH) by MPLC using the YMC C18 column to give 8 fractions (Fr. 1C-1-1C-8). The fraction 1C-2 was separated by a Sephadex LH-20 column and eluted by MeOH, and its subfraction was isolated by prep-HPLC to give compound 1 (460.0 mg; purity > 97%) and compound 2 (586.0 mg; purity > 95%).

### 2.12. Sample Preparation for HPLC Analysis

The standard stock solutions for HPLC were prepared by dissolving accurately weighed compounds in 100% methanol (1 mg/mL). SR extract was prepared at a concentration of 10 mg/mL. All solutions for analysis were filtered through 0.45 *μ*m RC membrane syringe filters (Sartorius, Germany).

### 2.13. Optimization of Chromatographic Conditions

HPLC analysis was performed using a Dionex UltiMate 3000 system (Dionex Corp., Sunnyvale, CA, USA) equipped with a binary pump, autosampler, column oven, and diode array UV/VIS detector (DAD). System control and data analysis were carried out using Dionex Chromeleon software. Separation was carried out on a Luna C18 column (250 × 4.6 mm, 5 *μ*m, Phenomenex, Torrance, CA, USA), with the column oven temperature kept at 30°C, at a UV wavelength of 200 nm. The mobile phase consisted of water (solvent A) and methanol (solvent B) with 25 : 75 at 0–20 min, 75%–100% of B at 20-30 min in a flow rate of 1.0 mL/min.

### 2.14. Validation of the Method

The linear calibration curves were plotted with diluted five different concentrations. Linearity was assessed by computing the correlation coefficient (*R*^2^) of the calibration curve for two compounds. Five concentrations of compounds were analyzed in triplicate. Regression equations were calculated using the equation *y* = *ax* ± *b*, where *x* and *y* are the concentration and peak area of the compound, respectively.

### 2.15. Statistical Analyses

All data are presented as the mean ± standard error of the mean of three independent experiments. Statistical significance was analyzed using one-way analysis of variance followed by Dunnett's test after comparing the LPS and each treated sample. Statistical significance was defined as *p* < 0.05.

## 3. Results

### 3.1. Influence of SR on the Viability of BV2 Cells

**Figure 1:**
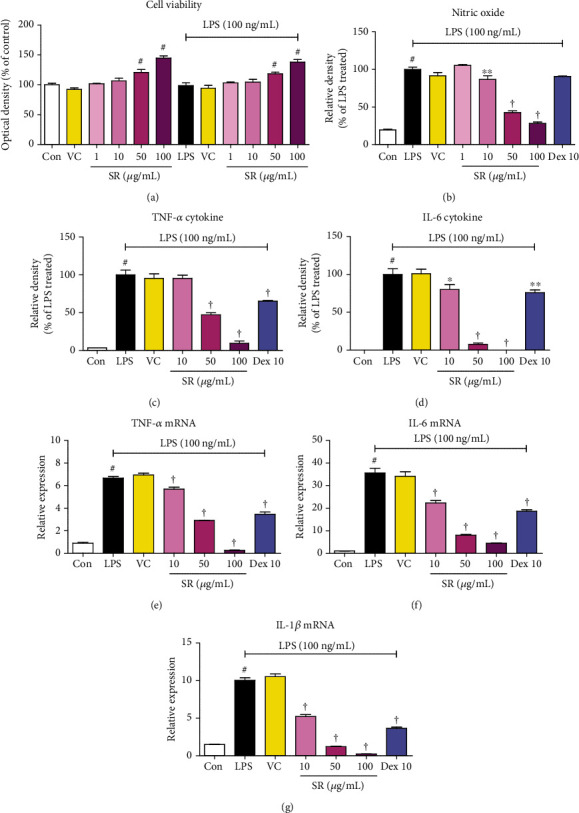
Effects of Saussureae Radix (SR) on (a) viability of microglia, secretion of (b) nitric oxide (NO) and (c, d) inflammatory cytokines, and (e–g) expression of cytokine mRNA in BV2 cells. Control cells were incubated with only fresh DMEM. BV2 cells were incubated with SR, vehicle control (VC; 0.1% dimethyl sulfoxide (DMSO)), or stimulated with lipopolysaccharide (LPS) for 24 (a–d) or 12 h (e–g). TNF: tumor necrosis factor; IL: interleukin. ^#^*p* < 0.05 (vs. control); ^∗^*p* < 0.05 and ^†^*p* < 0.001 (vs. LPS).

To examine the potential cytotoxic effects of SR on BV2 cells, CCK assays were conducted. As presented in Figure 1(a), treatment with SR (1–100 *μ*g/mL) for 24 h caused no cytotoxicity, and slight proliferation was observed at concentrations of ≥50 *μ*g/mL. Also, 0.1% DMSO used as a vehicle control had no significant effect on cell viability.

### 3.2. Effects of SR on the Secretion of NO and Cytokines

To investigate the antineuroinflammatory efficacy of SR, we first evaluated the NO production following LPS stimulation using Griess assay. As presented in Figure 1(b), LPS treatment strongly elevated NO levels, whereas pretreatment with SR strongly diminished NO production in a concentration-dependent manner. We next investigated the influences of SR on the LPS-induced inflammatory cytokine production and expression of their mRNAs using ELISA and RT-qPCR, respectively. As shown in Figures 1(c)–1(g), LPS-treated cells strongly increased the production of tumor necrosis factor- (TNF-) *α* and interleukin- (IL-) 6 as well as their mRNA levels, whereas SR treatment suppressed cytokine and mRNA levels in a concentration-dependent manner. In addition, treatment of 0.1% DMSO (vehicle control) did not affect the production of neuroinflammatory mediators.

### 3.3. Effects of SR on the Expression of iNOS and COX-2

**Figure 2:**
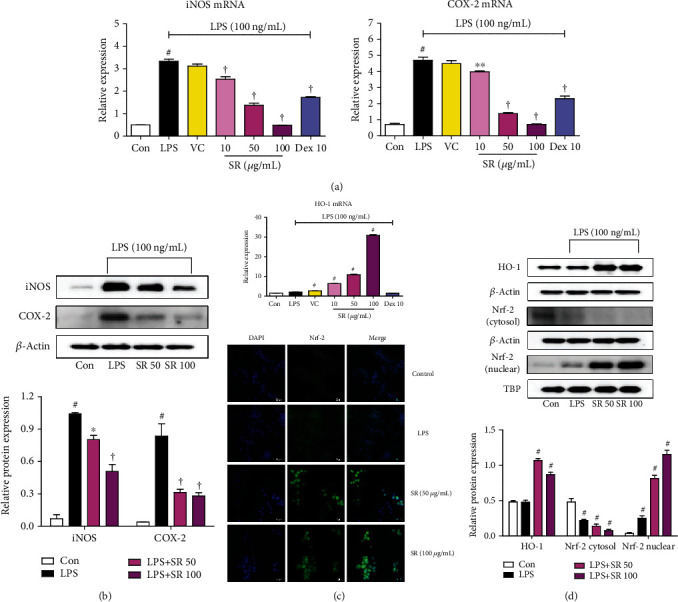
Effects of Saussureae Radix (SR) on (a, b) the expression of inducible nitric oxide synthase (iNOS) and cyclooxygenase- (COX-) 2, (c) the mRNA expression of heme oxygenase- (HO-) 1, and (d) the protein expression of HO-1 and nuclear translocation of nuclear factor erythroid 2-related factor 2 (Nrf-2). Control cells were incubated with only fresh DMEM. BV2 cells were stimulated with lipopolysaccharide (LPS) for 12 (iNOS and COX-2), 6 (HO-1 protein), or 3 h (HO-1 mRNA and Nrf-2). (b, d) The histogram graphs show protein expression levels relative to those of the housekeeping protein. (d) The cultured BV2 microglia were incubated with anti-Nrf-2 (green) and DAPI (blue). Fluorescence was developed using the Alexa Fluor 488-conjugated anti-rabbit secondary antibody. ^#^*p* < 0.05 (vs. control); ^∗^*p* < 0.05 and ^†^*p* < 0.001 (vs. LPS).

We next researched the protein and mRNA expression of iNOS and COX-2, the synthesizing enzymes of NO and prostaglandin E_2_, respectively. The results indicated that pretreatment with SR effectively suppressed LPS-induced iNOS and COX-2 expression at the protein and mRNA levels (Figures 2(a) and 2(b)).

### 3.4. Effects of SR on the Induction of HO-1 and Nuclear Translocation of Nrf-2

HO-1 was effectively induced by SR pretreatment even under LPS stimulation. HO-1 protein expression was strongly induced by ≥50 *μ*g/mL SR, and its mRNA expression was induced by 100 *μ*g/mL SR (Figures 2(c) and 2(d)). The nuclear translocation of Nrf-2, which is the molecular mechanism that regulates HO-1 induction, was also elevated by SR, and the extent of translocation was increased with increasing SR concentrations (Figure 2(d)). We also used immunofluorescence imaging to confirm the effect of SR on the nuclear translocation of Nrf-2 in BV2 cells and obtained results similar to those of Western blotting (Figure 2(d)).

### 3.5. Effects of SR on the Transcriptional Activity of NF-*κ*B

**Figure 3:**
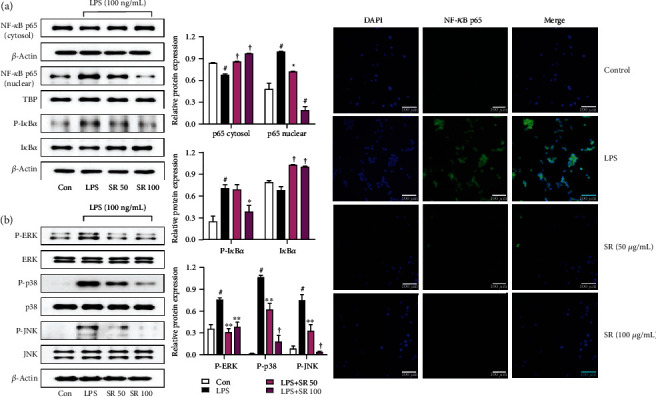
Effects of Saussureae Radix (SR) on the (a) nuclear translocation of nuclear factor- (NF-) *κ*B p65 and phosphorylation of inhibitor of NF-*κ*B alpha (I*κ*B*α*) and (b) phosphorylation of three mitogen-activated protein kinases (MAPKs). Control cells were incubated with only fresh DMEM. Cells were stimulated with lipopolysaccharide (LPS) for 1 h (NF-*κ*B p65) or 30 min (I*κ*B*α* and MAPK). The histogram graphs show protein expression levels relative to those of the housekeeping protein. (a) The cultured BV2 microglia were incubated with anti-NF-*κ*B p65 (green) and DAPI (blue). Fluorescence was developed using the Alexa Fluor 488-conjugated anti-rabbit secondary antibody. ^#^*p* < 0.05 (vs. control); ^∗^*p* < 0.05, ^∗∗^*p* < 0.01, and ^†^*p* < 0.001 (vs. LPS).

Because the NF-*κ*B pathway plays a pivotal role in the neuroinflammatory process, we examined the inhibitory efficacy of SR on the phosphorylation of I*κ*B*α* and NF-*κ*B transactivation in LPS-stimulated microglia. Western blotting revealed that SR effectively inhibited p65 translocation from the cytoplasm to the nucleus in a concentration-dependent manner (Figure 3(a)). Additionally, SR strongly decreased the phosphorylation of I*κ*B*α* upon LPS stimulation (Figure 3(a)). Moreover, the nuclear translocation of p65 as identified using immunofluorescence imaging was strongly suppressed by SR pretreatment, in line with the Western blotting result (Figure 3(a)).

### 3.6. Effects of SR on MAPK Phosphorylation

The phosphorylation of MAPK is involved in the production of many proinflammatory factors. Therefore, we analyzed the change of the phosphorylation of three MAPKs including extracellular signal-regulated kinase (ERK), p38, and c-Jun NH_2_-terminal kinase (JNK). Results of Figure 3(b) indicated that SR pretreatment repressed the activation of MAPKs, including ERK, p38, and JNK.

### 3.7. Effects of SR on LPS-Induced AP-1 Pathway Activation

**Figure 4:**
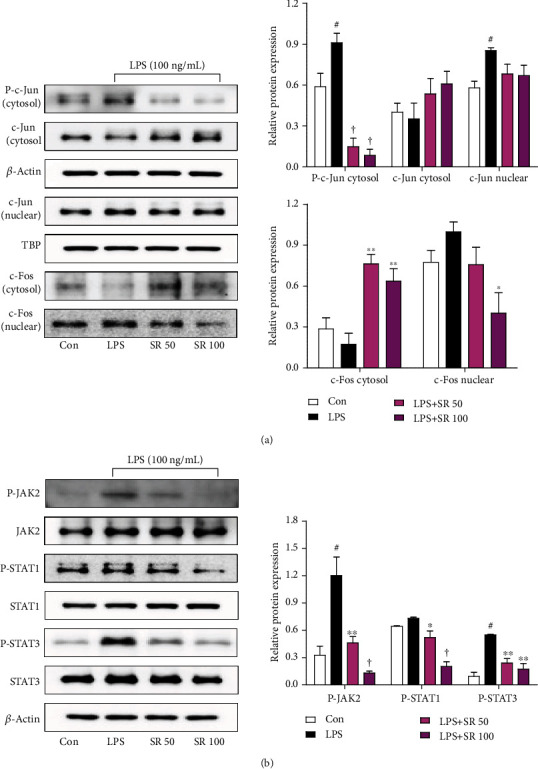
Effects of Saussureae Radix (SR) on the (a) nuclear translocation and phosphorylation of c-Jun and c-Fos and (b) phosphorylation of Janus kinase (JAK2), signal transducer and activator of transcription 1 (STAT1), and STAT3. Control cells were incubated with only fresh DMEM. Cells were stimulated with lipopolysaccharide (LPS) for (a) 1 or (b) 4 h. The histograms show protein levels relative to those of the internal control. ^#^*p* < 0.05 (vs. control); ^∗^*p* < 0.05, ^∗∗^*p* < 0.01, and ^†^*p* < 0.001 (vs. LPS).

AP-1 migrates to the nucleus and regulates the expression of certain inflammatory genes in response to inflammation caused by stimuli, such as LPS [6]. We therefore measured the level of AP-1 phosphorylation in the cytoplasm and nuclear AP-1 levels. As presented in Figure 4(a), LPS stimulation induced c-Jun phosphorylation and its migration to the nucleus, whereas pretreatment with SR inhibited the nuclear transfer and phosphorylation of c-Jun. The nuclear translocation of c-Fos, another subunit of AP-1, was also suppressed in a similar manner by SR pretreatment (Figure 4(a)).

### 3.8. Effects of SR on Activation of the JAK/STAT Pathway

Previous research reported that mitigation of the JAK/STAT pathway inhibited the secretion of NO and proinflammatory cytokines [18]. Therefore, we measured the inhibitory effects of SR on the phosphorylation of JAK2, STAT1, and STAT3 in LPS-stimulated BV2 cells. As presented in Figure 4(b), SR treatment significantly blocked the phosphorylation of JAK2, STAT1, and STAT3 without affecting their total protein levels.

### 3.9. Isolation and Structural Elucidation

**Figure 5:**
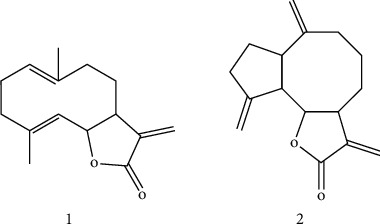
Structure of costunolide (1) and dehydrocostuslactone (2) isolated from SR.

Using combined chromatographic separation techniques, costunolide (1) and dehydrocostuslactone (2) were effectively isolated from SR (Figure 5). The HPLC evaluation showed that the purity of the isolated compounds was >95%. The structures were confirmed by comparing the obtained spectroscopic data with the previously reported values [19].

### 3.10. Verification of Antineuroinflammatory Effects of Costunolide and Dehydrocostuslactone in BV2 Microglia upon LPS Stimulation

**Figure 6:**
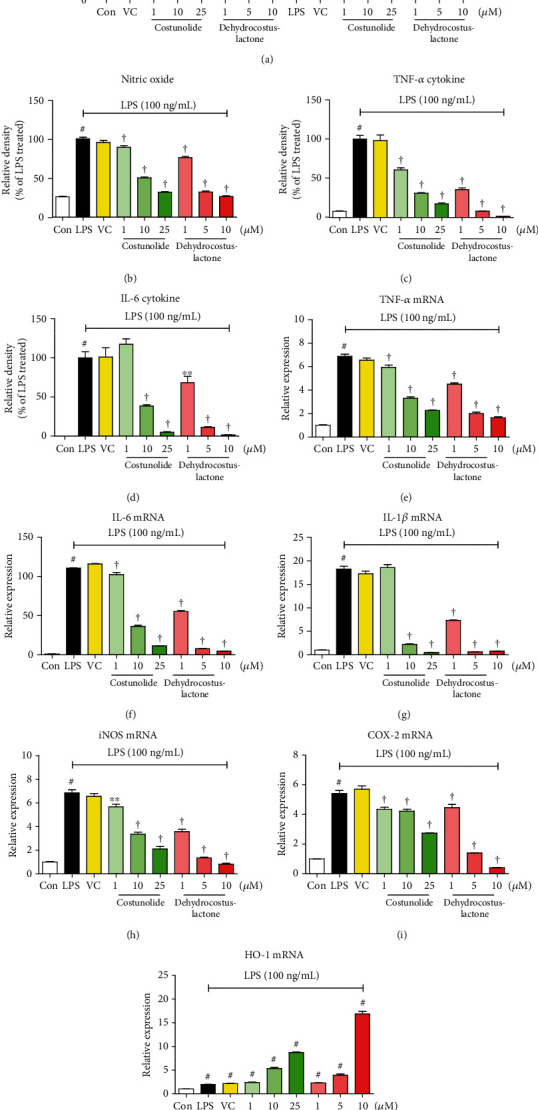
Effects of two compounds costunolide and dehydrocostuslactone on (a) viability of microglia, secretion of (b) nitric oxide (NO) and (c, d) inflammatory cytokines, and expression of (e–g) cytokine mRNAs, (h, i) inducible nitric oxide synthase (iNOS) and cyclooxygenase- (COX-) 2 mRNAs, and (j) heme oxygenase- (HO-) 1 mRNA. Control cells were incubated with only fresh DMEM. BV2 cells were incubated with each compound, 0.1% dimethyl sulfoxide (DMSO), or stimulated with lipopolysaccharide (LPS) for 24 (a, b), 6 (c–g), 3 (h, i), or 6 h (j). ^#^*p* < 0.05 (vs. control); ^∗^*p* < 0.05, ^∗∗^*p* < 0.01, and ^†^*p* < 0.001 (vs. LPS).

To investigate the cytotoxicity of the main constituents of SR, we carried out CCK assays on microglia. As shown in Figure 6(a), costunolide showed no cytotoxicity at concentrations below 25 *μ*M and dehydrocostuslactone at concentrations below 10 *μ*M. The two components showed slight cytotoxicity at concentrations greater than 25 *μ*M and 10 *μ*M, respectively, so subsequent experiments used only concentrations below that. Also, both costunolide and dehydrocostuslactone effectively suppressed the production of NO and cytokines induced by LPS stimulation (Figures 6(b)–6(d)), and each mRNA expression was significantly and concentration-dependently suppressed (Figures 6(e)–6(g)). In addition, as shown in Figures 6(h)–6(j), the mRNA levels of iNOS and COX-2 were strongly suppressed by the pretreatment of costunolide and dehydrocostuslactone, and the expression of the antioxidant enzyme HO-1 mRNA was effectively induced. The 0.1% DMSO used as a vehicle control had no effect on cell viability and the production of neuroinflammatory mediators. In most of the above results, costunolide and dehydrocostuslactone showed concentration-dependent effects with statistical significance, respectively.

### 3.11. Identification of the Components of SR Using HPLC-DAD Analysis

**Figure 7:**
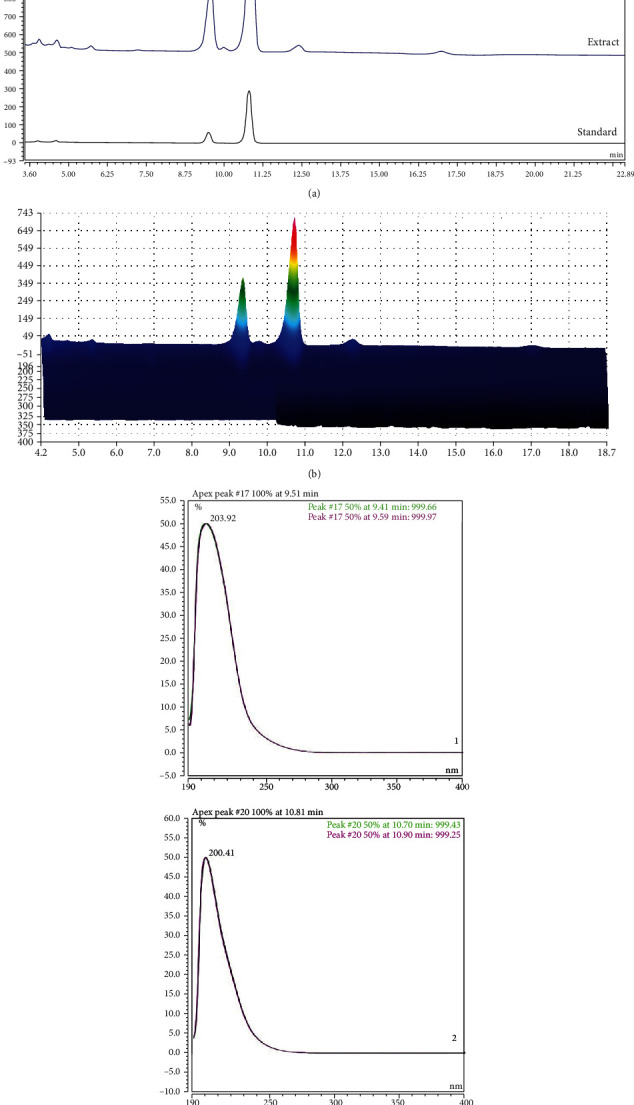
HPLC chromatograms ((a) 2D; (b) 3D) of two standard compounds from the ethanolic extract of SR at UV wavelengths of 200 nm (c). Costunolide (1) (9.39 min) and dehydrocostuslactone (2) (10.68 min) were identified.

The mobile phase consisted of methanol and water. Two standards and SR were detected using a PDA detector (200-400 nm). A wavelength of 200 nm was selected according to their maximum wavelength. By comparison of retention times (tR) and UV spectra of two standards (costunolide (1) (9.39 min) and dehydrocostuslactone (2) (10.68 min)) and SR, two compounds were detected (Figure 7).

### 3.12. Validation of the Analytical HPLC Method

The calibration curves for two compounds were obtained via plotting the peak area versus the concentration. The linear correlation coefficient (*R*^2^) for the calibration curves was greater than 0.999 (Table 3). According to the calibration curves, the amounts of two compounds were found to be 62.81996 ± 0.0186 and 19.63455 ± 0.0042 mg/g, respectively.

## 4. Discussion

Microglial cell-mediated neuroinflammation has been identified as an important risk factor for neurodegenerative diseases. Overactivation of microglial cells leads to neuronal damage, brain injury, and the release of various neuroinflammatory mediators [20]. Reducing levels of these endogenous inflammatory factors through regulation of microglial activation is important in preventing and treating neuroinflammation [21]. SR is the dried root of *Saussurea lappa* Clarke (Compositae) and is native to the Himalayas in India. SR is listed in the ancient medicine text “Shennong's Classic of Materia Medica” and has long been used in East Asia to treat digestive disorders such as indigestion, vomiting, stomachache, diarrhea, and chronic inflammation of the digestive system. SR has also been used in the treatment of dysentery and testicular inflammation, and some recent studies have shown that SR can ameliorate reproductive toxicity in rats and alleviate house dust mite-induced atopic dermatitis in mice [12, 13]. In addition, a recent review study described that SR exhibits anticancer, anti-inflammatory, antiulcer, and cholagogic effects [14]. Furthermore, another recent study shows that SR has antioxidant and antineuroinflammatory efficacy [22]. However, the above work used ethyl acetate fraction of SR and demonstrated inhibitory activity for NO, iNOS, and TNF-*α* cytokine production along with 2,2-diphenyl-1-picrylhydrazyl radical scavenging activity. Our present study demonstrated the inhibitory activity of SR on the generation of neuroinflammatory factors such as IL-6, IL-1*β*, and COX-2 as well as NO in BV2 cells activated by LPS stimulation. We also revealed the molecular mechanisms of antineuroinflammatory activity through investigating the effects of SR on the NF-*κ*B, MAPK, AP-1, and JAK/STAT pathways. Additionally, we have identified the efficacy of SR for activation of the HO-1/Nrf-2 antioxidant pathway.

First of all, we conducted a CCK assay to exclude the potential cytotoxicity of SR to BV2 microglial cells, and SR did not affect cell viability up to 100 *μ*g/mL. Furthermore, the proliferation of BV2 cells was confirmed when more than 50 *μ*g/mL of SR was treated (Figure 1(a)). NO is a free radical that has been involved in the microglial cell-mediated inflammatory process in the CNS [23]. NO is synthesized from L-arginine by iNOS, and overproduction of NO is related to the presence of some inflammatory diseases and autoimmune disorders [24, 25]. NO secretion and iNOS expression levels were closely related to HO-1 induction [26], and HO-1 is regulated by Nrf-2. We therefore examined whether SR pretreatment inhibited the production of NO, inflammatory cytokines, iNOS, and COX-2 during the neuroinflammatory process upon LPS stimulation in microglial cells. Results of these experiments demonstrated that SR significantly reduced the production of NO, inflammatory cytokines, and its mRNAs without cytotoxicity (Figure 1) and inhibited the expression of iNOS and COX-2 protein and mRNA with statistical significance (Figures 2(a) and 2(b)). We further examined the effect of SR treatment on the induction of HO-1 and activation of Nrf-2. We found that SR markedly induced HO-1 expression and gradually increased the nuclear translocation of Nrf-2 with statistical significance (Figures 2(c) and 2(d)). The efficacy of SR for HO-1 induction and Nrf-2 activation appears to have an indirect effect on regulating the neuroinflammatory response by inhibiting NO and iNOS.

The activation of NF-*κ*B and MAPK is associated with the pathogenesis in various diseases of CNS. NF-*κ*B is the main regulator of various genes involved in immune and inflammatory reactions [27]. Depending on the activation of microglia by LPS, I*κ*B*α* is degraded and phosphorylated, in which free NF-*κ*B is moved to the nucleus and activates inflammatory mediators [28]. MAPK plays an important role in the expression induced by LPS of some endogenous inflammatory mediators and the activation of NF-*κ*B [29, 30]. We examined whether the influence of SR on inflammatory mediators is associated with the change of NF-*κ*B. Our results showed that treatment of SR efficiently repressed the nuclear translocation of NF-*κ*B p65 in a concentration-dependent manner (Figure 3(a)). In addition, treatment of SR inhibited the phosphorylation and degradation of I*κ*B*α* in a similar pattern (Figure 3(a)). We also investigated the effects of SR on the phosphorylation of MAPK proteins following LPS stimulation, revealing that MAPK activation was effectively suppressed by SR treatment through inhibiting phosphorylation of ERK, p38, and JNK (Figure 3(b)). These results indicated that the inhibitory effects of SR on neuroinflammation might be controlled via the blockade of the activation of NF-*κ*B and MAPK pathways.

The activation and nuclear translocation of AP-1 subunits regulate the expression of some inflammatory genes in inflammatory responses induced by LPS [6]. The JAK/STAT pathway is known to elicit the production of various inflammatory mediators through phosphorylation and is recognized to have a central role in inflammatory responses [6, 31]. In addition, the phosphorylation of STAT3 has a direct effect on IL-6 secretion [32]. We therefore examined the effects of SR on the phosphorylation of c-Jun and nuclear transfer of c-Jun and c-Fos following LPS treatment, and the results demonstrated that SR pretreatment inhibited both phosphorylation and nuclear translocation of c-Jun and suppressed translocation into the nucleus of c-Fos (Figure 4(a)). In addition, SR pretreatment effectively inhibited the phosphorylation of JAK2, STAT1, and STAT3 (Figure 4(b)). These results indicated that the antineuroinflammatory activity of SR was attributable to both the blockade of the NF-*κ*B and MAPK pathways and the inhibition of the AP-1 and JAK/STAT pathways.

We also identified the two main components (costunolide and dehydrocostuslactone) of the SR using HPLC analysis. Previous studies of these components have shown that costunolide ameliorates acute lung injury via attenuating MAPK and improved acute ulcerative colitis in mice through inactivation of NF-*κ*B, STAT1/3, and Akt [33, 34]. There have also been reports that dehydrocostuslactone suppresses LPS-induced macrophage activation through NF-*κ*B, p38 MAPK, and Akt [35]. Also, dehydrocostuslactone has been researched to be effective in inhibiting LPS-induced inflammation *in vitro* and improves the survival of mice in cecal ligation and puncture-induced sepsis *in vivo* [36]. These studies indicated that the antineuroinflammatory effect of SR is closely connected with the efficacy of its constituents, costunolide and dehydrocostuslactone. To confirm the antineuroinflammatory activity of these compounds, we investigated the effect of costunolide and dehydrocostuslactone on the production of NO, proinflammatory cytokines, inflammatory mRNAs, and antioxidant enzyme HO-1 on the microglia. Our results indicated that these compounds inhibited the production of NO and inflammatory cytokines including TNF-*α* and IL-6 without cytotoxicity at each concentration (Figures 6(a)–6(d)). Also, two compounds suppressed the expression of cytokine, iNOS, and COX-2 mRNA in a concentration-dependent manner (Figures 6(e)–6(i)). In addition, costunolide and dehydrocostuslactone strongly induced HO-1 mRNA expression (Figure 6(j)). Based on the above results, the regulatory efficacy of SR on the neuroinflammatory response is thought to be closely related to the inhibitory efficacy of costunolide and dehydrocostuslactone on the production of inflammatory mediators.

The results of this study showed that SR contains antineuroinflammatory properties *in vitro* by attenuating inhibiting inflammatory mediators in LPS-stimulated BV2 cells. These beneficial activities are associated with enhanced HO-1/Nrf-2 activation and inhibition of the NF-*κ*B/MAPK/AP-1/JAK/STAT signaling pathways. The antineuroinflammatory effects of SR appear to be closely related to the presence of two components, costunolide and dehydrocostuslactone. Based on these results, SR appears to have potential value as a candidate for the treatment of inflammation-related degenerative brain diseases.

## Figures and Tables

**Table 1:** Primers used for RT-qPCR.

Target gene	Primer sequence
TNF-*α*	F: 5′-TTCTGTCTACTGAACTTCGGGGTGATCGGTCC-3′
	R: 5′-GTATGAGATAGCAAATCGGCTGACGGTGTGGG-3′
IL-6	F: 5′-TCCAGTTGCCTTCTTGGGAC-3′
	R: 5′-GTGTAATTAAGCCTCCGACTTG-3′
IL-1*β*	F: 5′-ATGGCAACTGTTCCTGAACTCAACT-3′
	R: 5′-CAGGACAGGTATAGATTCTTTCCTTT-3′
iNOS	F: 5′-GGCAGCCTGTGAGACCTTTG-3′
	R: 5′-GCATTGGAAGTGAAGCGTTTC-3′
COX-2	F: 5′-TGAGTACCGCAAACGCTTCTC-3′
	R: 5′-TGGACGAGGTTTTTCCACCAG-3′
HO-1	F: 5′-TGAAGGAGGCCACCAAGGAGG-3′
	R: 5′-AGAGGTCACCCAGGTAGCGGG-3′
*β*-Actin	F: 5′-AGAGGGAAATCGTGCGTGAC-3′
	R: 5′-CAATAGTGATGACCTGGCCGT-3′

F: forward; R: reverse.

**Table 2:** Primary and secondary antibodies used for Western blotting analysis.

Antibody	Corporation	Product no.	RRID	Dilution rate
iNOS	Cell Signaling	#13120	AB_2687529	1 : 1000
COX-2	Cell Signaling	#4842	AB_2085144	1 : 1000
*β*-Actin	Santa Cruz	#SC-47778	AB_626632	1 : 1000
HO-1	Cell Signaling	#82206	AB_2799989	1 : 1000
Nrf-2	Novus Biologicals	#NBP1-32822	AB_10003994	1 : 1000
TBP	Cell Signaling	#8515	AB_10949159	1 : 1000
NF-*κ*B p65	Cell Signaling	#8242	AB_10859369	1 : 1000
P-I*κ*B*α*	Cell Signaling	#2859	AB_561111	1 : 1000
I*κ*B*α*	Cell Signaling	#4814	AB_390781	1 : 1000
P-ERK	Cell Signaling	#4377	AB_331775	1 : 1000
ERK	Cell Signaling	#9102	AB_330744	1 : 1000
P-p38	Cell Signaling	#9211	AB_331641	1 : 1000
p38	Cell Signaling	#9212	AB_330713	1 : 1000
P-JNK	Cell Signaling	#9251	AB_331659	1 : 1000
JNK	Cell Signaling	#9252	AB_2250373	1 : 1000
P-c-Jun	Cell Signaling	#3270	AB_2129575	1 : 1000
c-Jun	Cell Signaling	#9165	AB_2130165	1 : 1000
c-Fos	Cell Signaling	#2250	AB_2247211	1 : 1000
P-JAK2	Cell Signaling	3771	AB_330403	1 : 1000
JAK2	Cell Signaling	3230	AB_2128522	1 : 1000
P-STAT1	Cell Signaling	9167	AB_561284	1 : 1000
STAT1	Cell Signaling	14994	AB_2737027	1 : 1000
P-STAT3	Cell Signaling	9145	AB_2491009	1 : 1000
STAT3	Cell Signaling	12640	AB_2629499	1 : 1000
2nd anti-mouse	Cell Signaling	#7076	AB_330924	1 : 5000
2nd anti-rabbit	Cell Signaling	#7074	AB_2099233	1 : 5000

**Table 3:** Regression data and contents of two compounds in SR.

Analytes	Regression equation	*R* ^2^	Content (mg/g)
Costunolide (1)	*y* = 0.173*x* + 0.0257	0.9999	62.81996 ± 0.0186
Dehydrocostuslactone (2)	*y* = 1.0619*x* + 9.5492	0.9994	19.63455 ± 0.0042

## Data Availability

The data used to support the findings are available from the corresponding author upon request.

## Abbreviations

AP: Activator protein
BSA: Bovine serum albumin
CCK: Cell-counting kit
CNS: Central nervous system
CO: Carbon monoxide
COX: Cyclooxygenase
DAD: Diode array UV/VIS detector
DAPI: 4,6-Diamidino-2-phenylindole
DMEM: Dulbecco's modified Eagle's medium
DMSO: Dimethyl sulfoxide
ELISA: Enzyme-linked immunosorbent assay
ERK: Extracellular signal-regulated kinase
FBS: Fetal bovine serum
HO: Heme oxygenase
HPLC: High-performance liquid chromatography
HRP: Horseradish peroxidase
IL: Interleukin
iNOS: Inducible nitric oxide synthase
JAK: Janus kinase
JNK: c-Jun NH_2_-terminal kinase
LPS: Lipopolysaccharide
MAPK: Mitogen-activated protein kinase
NF: Nuclear factor
NO: Nitric oxide
Nrf-2: Nuclear factor erythroid 2-related factor 2
PBS: Phosphate-buffered saline
RT-qPCR: Real-time reverse transcription-polymerase chain reaction
SR: Saussureae Radix
STAT: Signal transducer and activator of transcription
TNF: Tumor necrosis factor
VC: Vehicle control.
